# High-temperature heat pumps: market overview, state of the art, and application potential

**DOI:** 10.1007/s10098-026-03452-5

**Published:** 2026-05-13

**Authors:** Cordin Arpagaus

**Affiliations:** OST – Eastern Switzerland, University of Applied Sciences, Institute for Energy Systems, Buchs, Switzerland

**Keywords:** High-temperature heat pumps, Industrial process heat, Electrification, Refrigerants, Energy efficiency, Decarbonization, Heat pump technology

## Abstract

High-temperature heat pumps (HTHPs) are a key technology for enabling the decarbonization of industrial process heat up to about 200 °C and for generating steam. This paper reviews the state of the art in HTHP technologies, presents case studies from Europe, and discusses their potential applications in New Zealand. Industrial sectors such as dairy, food processing, and chemicals are identified as particularly relevant due to their substantial demand for low- to medium-temperature heat. Case studies from European demonstration projects demonstrate the successful integration of HTHP, achieving coefficients of performance (COP) ranging from 2.5 to 4.5 under varying operating conditions, while delivering significant energy savings and CO₂ emission reductions. The transition to natural refrigerants, large-scale demonstrations, and supportive policy frameworks emerge as critical enablers. Economic feasibility is strongly influenced by market dynamics, particularly the ratio of electricity to fossil-fuel prices and the CO₂ intensity of the electricity supply. Environmental regulations also significantly influence refrigerant availability and system design choices. Technical challenges remain in compressor design, refrigerant selection, and efficiency limitations associated with large temperature lifts. The analysis highlights three priorities for accelerating market adoption: (A) greater dissemination of knowledge about the integration of HTHPs in industrial processes, (A) increasing the availability of low-global-warming potential (low-GWP) refrigerants, and (B) improving the cost-competitiveness of electricity relative to fossil fuels. With these developments, HTHPs can play a central role in sustainable industrial energy transitions.

**Graphical Abstract:**

**Figure:**
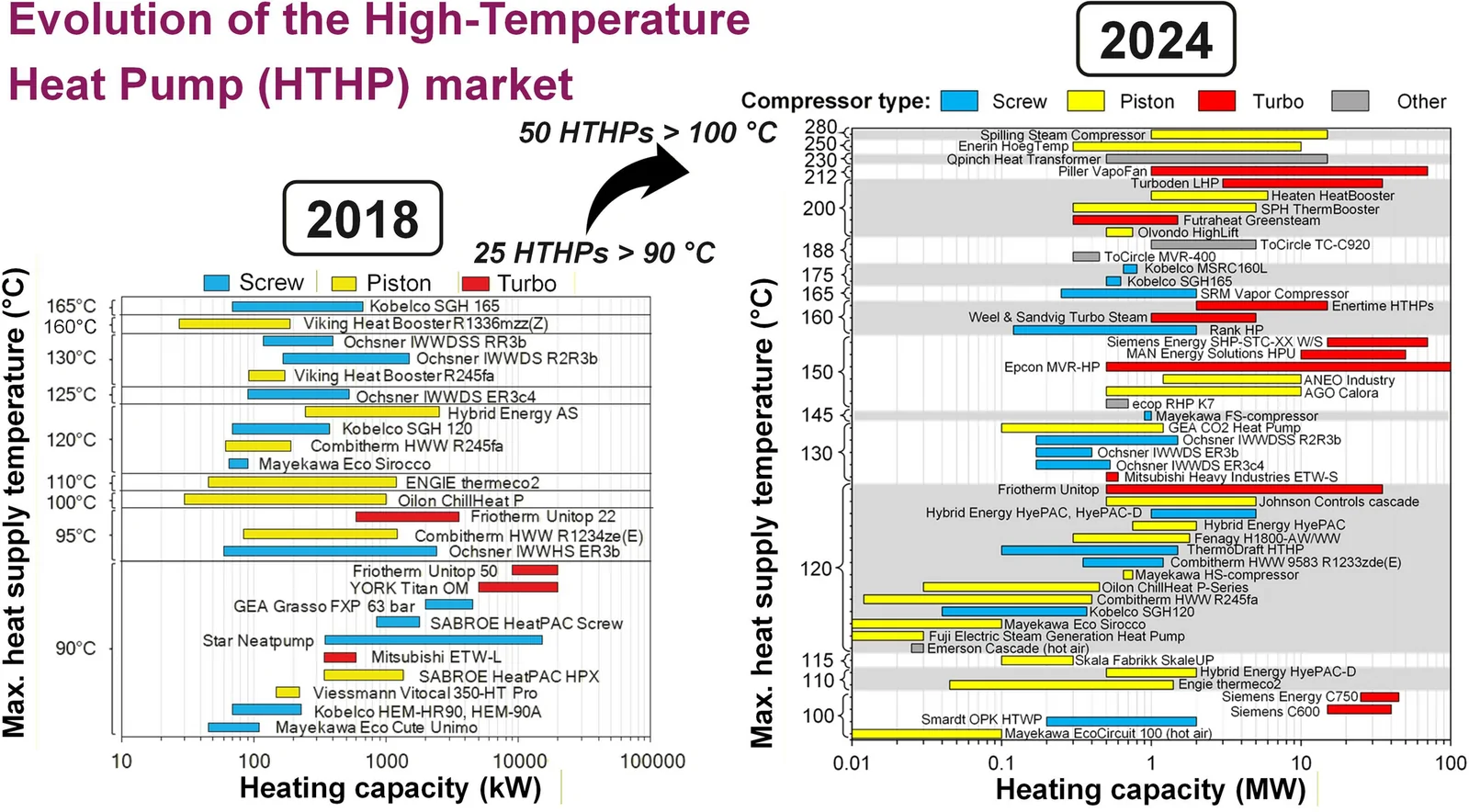
Evolution of the high-temperature heat pump (HTHP) market from 2018 to 2024, showing the growing number of HTHP suppliers, categorized by maximum heat supply temperature, heating capacity (logarithmic), and compressor technology (colors) (Figure credit: C. Arpagaus, OST)

## Introduction

### Relevance to the special issue theme

This manuscript addresses the Special Issue’s focus on scalable, low-carbon industrial energy solutions by analyzing HTHPs as a key technology for decarbonizing industrial process heat. HTHPs uniquely combine energy efficiency, electrification, and waste-heat utilization at temperatures typically served by fossil fuels.

The study reviews the evolution of the HTHP market, steam-generating heat pump concepts, achievable COPs, refrigerants, and European demonstration projects, complemented by an excursus on advanced adoption in New Zealand’s food sector. The results identify feasible temperature ranges, application sectors, and system configurations where HTHPs can effectively replace conventional boilers, reduce CO₂ emissions, and support the transition to a climate-neutral industry, providing relevant insights for both industrial decision-makers and policy frameworks.

Building on recent applied research and international collaboration, this study synthesizes recent insights from European demonstration projects, industrial case studies, and techno-economic analyses to assess the application potential, technological readiness, cost aspects, and policy environments for wider HTHP deployment where fossil fuels still dominate industrial process heat supply (De Boer et al. 2020; Arpagaus 2025a, 2025b).

A dedicated excursus on the New Zealand context is included following the paper’s presentation at ICOSSE 25 (International Congress on Sustainability Science & Engineering, 20–22 February 2025, Auckland). This section consolidates insights into advancing HTHP adoption in New Zealand’s food sector and links international experience to regional decarbonization pathways.

### Methods and review approach

This review adopts a structured, evidence-based approach to assess the potential of HTHPs for industrial decarbonization. The scope covers industrial sectors with medium-to high-temperature process heat demand (up to 280 °C) and includes technologies, applications, market developments, and policy frameworks relevant to electrification of industrial heat.

Inclusion criteria were peer-reviewed articles, conference proceedings, demonstration project reports, and industrial case studies published between 2018 and 2025 that address HTHP suppliers, efficiency, techno-economic aspects, or adoption pathways. Data sources included international databases (Scopus, Web of Science), industry reports (EHPA, IEA, UNIDO), and government statistics (Swiss Federal Statistical Office), complemented by expert publications and recent demonstration project data from Europe and New Zealand.

This approach aligns with the Special Issue’s emphasis on scalable, low-carbon industrial energy solutions by synthesizing evidence on HTHP performance, market readiness, and adoption barriers, providing actionable insights for both industrial stakeholders and policymakers.

### Evolution of the HTHP market

Industry is a major contributor to global greenhouse gas (GHG) emissions, accounting for nearly 22% of direct emissions and approximately 31% of total emissions when indirect emissions from electricity use are included (UNIDO 2024). According to the IEA (2023), over 70% of industrial energy demand is still met by fossil fuels, mainly gas, oil, and coal. Decarbonizing this sector is crucial and requires comprehensive electrification strategies. Installing heat pumps will be a key milestone on the road to net zero. In the IEA’s Net-Zero Emissions by 2050 Scenario, approximately 500 MW of heat pump capacity must be installed every month for the next 30 years to meet 30% of industrial heat demand by 2050 (IEA 2021, 2022, 2024).

**Fig. 1:**
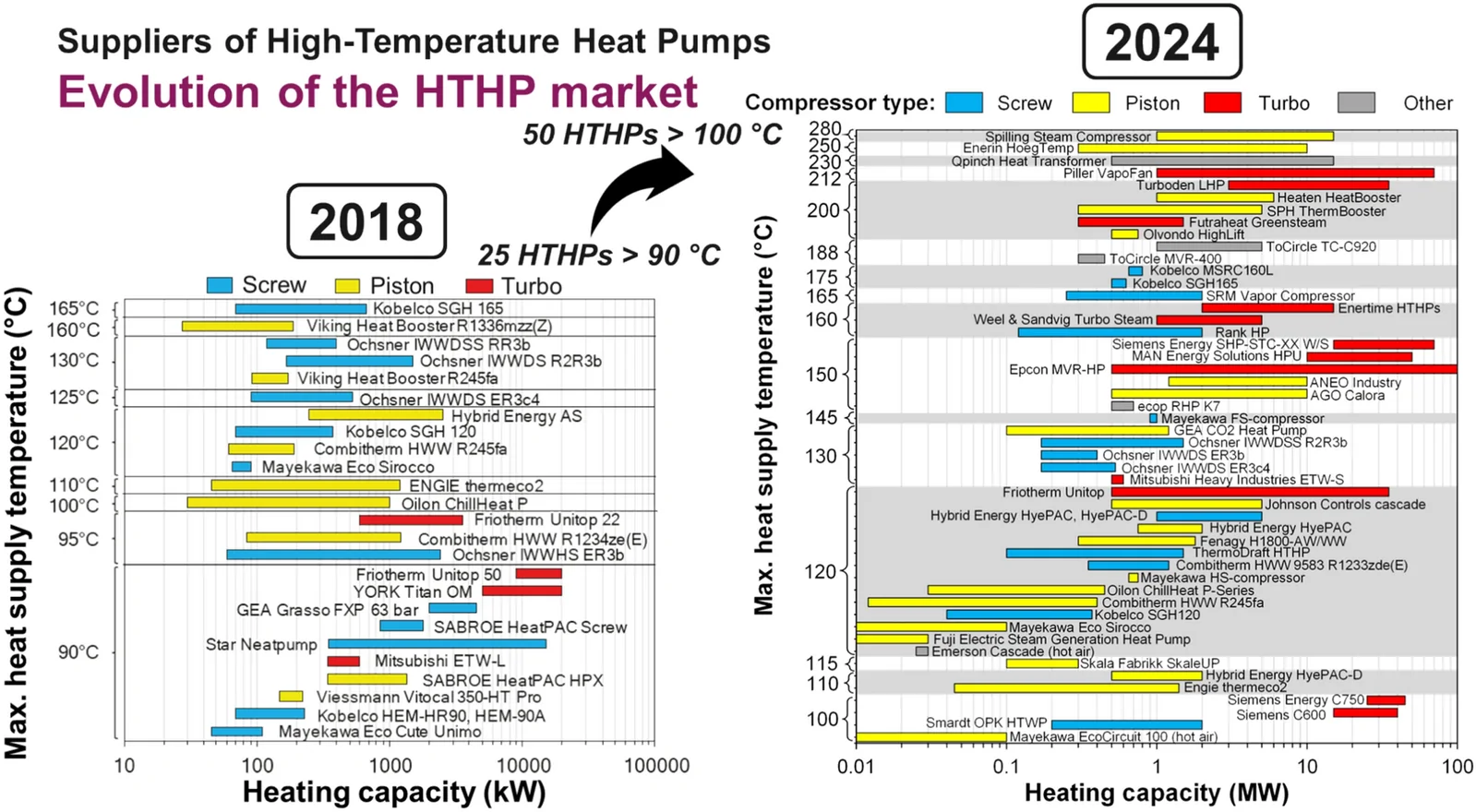
Evolution of the HTHP market, showing the growing number of HTHP suppliers, categorized by maximum heat supply temperature, heating capacity (logarithmic), and compressor technology (colors) (Arpagaus 2025a)

Industrial-scale high-temperature heat pumps (HTHPs) are emerging as a key solution for delivering efficient, low-emission heat in industrial processes (Arpagaus et al. 2018). Figure 1 illustrates the growth of the HTHP market, driven by the increasing number of suppliers (Arpagaus 2025a). Capable of supplying heat up to about 280 °C, HTHPs are vital for decarbonizing medium-temperature industrial heat demand.

Lessons learned from the residential sector highlight the pathway. In Switzerland, 74% of new residential buildings constructed between 2011 and 2024 used heat pumps (Swiss Federal Statistical Office 2024), exceeding the European average of around 28% for space-heating sales in 2024 (EHPA 2025). Other countries, such as Norway, Finland, and Sweden, continue to lead the European market, with heat pump space-heating share over 90% in 2024 (EHPA 2025).

This success, combined with extensive research and industrial experience, supports the upscaling of heat pump technologies for industrial applications (Obrist et al. 2023). Electrification of industrial process heat generation through HTHPs offers a promising pathway for decarbonization. However, scaling HTHP technology to meet larger-capacity and higher-temperature operation remains both technologically and economically challenging (Kosmadakis et al. 2020; Vieren et al. 2021; Jouhara et al. 2024; Arpagaus 2025b).

## HTHP technology status and key challenges

### COP, electricity-to-gas price ratio, and HTHP cycles

**Fig. 2:**
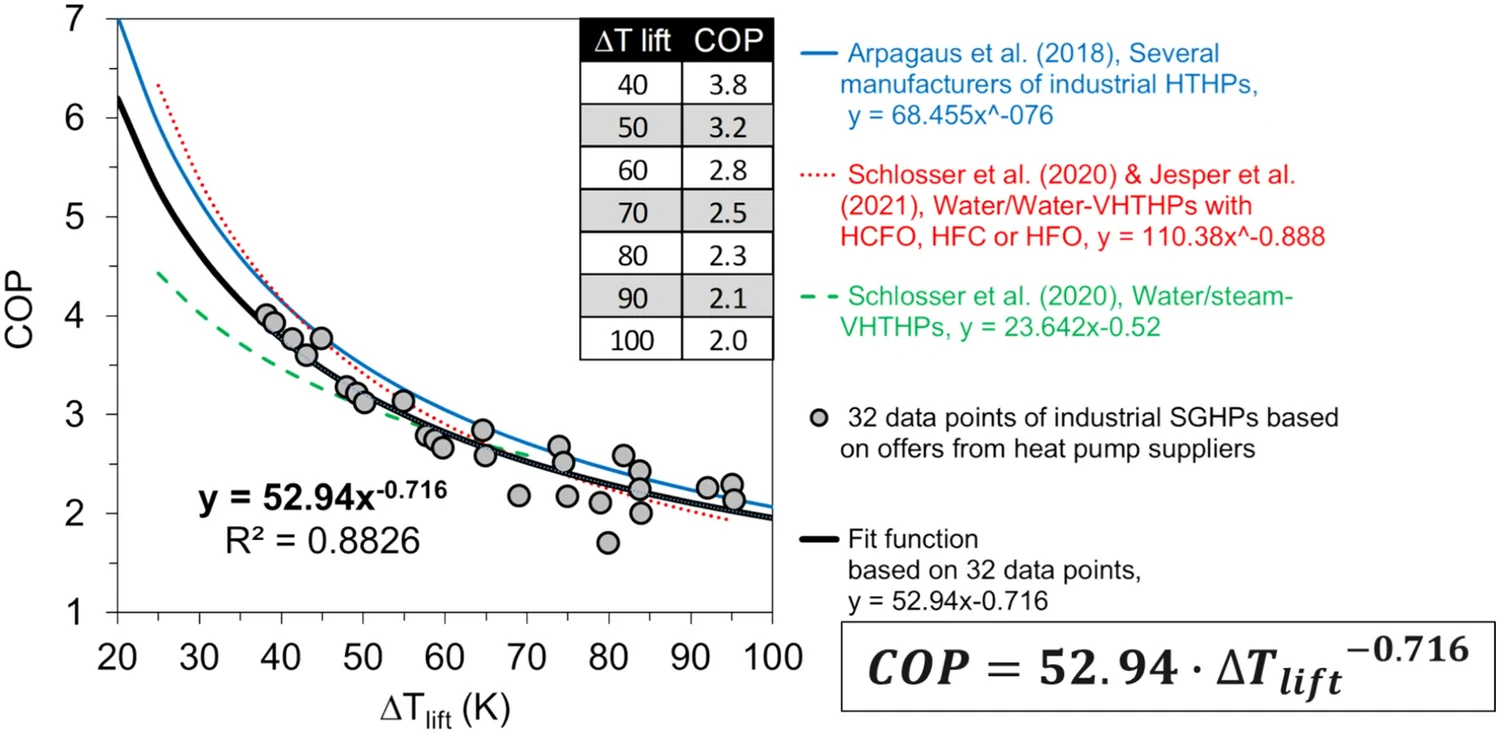
COP of steam-generating heat pumps (SGHPs) as a function of temperature lift illustrating efficiency trade-offs (Arpagaus et al. 2022). For comparison, some COP fit curves from the literature are shown (Arpagaus et al. 2018; Schlosser et al. 2020; Jesper et al. 2021)

The successful application of HTHPs depends largely on the temperature lift, i.e., the difference between the heat-source temperature (typically 30 °C–50 °C waste-heat streams) and the required process temperature (Arpagaus et al. 2022). Ensuring a temperature lift below 100 K is crucial to maintaining a coefficient of performance (COP) above 2.0 (Fig. 2), the minimum for economic viability given the electricity-to-gas price ratio.

Across European countries, the electricity-to-gas price ratio for small-scale industrial consumers ranges from roughly 1–5 (De Boer et al. 2020), strongly influencing the economic viability of electrified heating technologies. Market attractiveness is highly sensitive to this ratio: high electricity prices relative to gas undermine the business case for electrically driven HTHPs. Although expanded us of renewable electricity is essential for meeting decarbonization targets, electricity remains more expensive than fossil fuels in many European markets, creating a structural barrier to industrial fuel switching. Coal-dependent electricity grids require an HTHP COP above 4 to achieve net emissions reductions compared to direct fossil-fuel heating.

Figure 2 shows the COP as a function of the temperature lift between the heat-source inlet and the heat sink outlet. The COP is defined as the useful heat generated divided by the electrical power of the compressor. Typical COP values range from 2.5 to 3.8 for temperature lifts of 40–70 K, declining asymptotically toward 1 as the temperature lift exceeds 120 K. The formula $\left( {{\mathrm{COP}}\, = \,52.94 \cdot \Delta T_{{{\mathrm{Lift}}}}^{ - 0.716} } \right)$ represents a COP fit based on 32 data points from industrial SGHPs, based on offers from European heat pump suppliers. On average, an SGHP system achieves a Carnot efficiency of around 40–60% (Arpagaus et al. 2018).

This performance characteristic highlights the importance of carefully designing applications with industrial heat pumps to maximize efficiency. Stirling heat pumps achieve the highest temperature lift, followed by MVR applications. Cases with MVRs achieve higher efficiency than closed-cycle heat pumps due to their open-mode operation and efficient compressors. In the lower temperature lift ranges, closed-cycle heat pumps are the predominant technology.

The basic electrical-driven HTHP cycle comprises a compressor, condenser, expansion valve, and evaporator, utilizing refrigerants as working fluids. Key components include screw, piston, and turbo compressors adapted for higher discharge temperatures; oils and seals capable of withstanding temperatures exceeding 200 °C; and robust heat exchangers, such as shell-and-tube or plate designs, optimized for high-pressure steam. The primary design challenge is to ensure temperature resistance and minimize thermal losses. Material durability at high temperatures, compressor design for stable operation, lubricant efficacy, and safety measures related to flammable refrigerants are ongoing challenges. System insulation and mitigation of radiative heat losses are crucial for preserving efficiency.

**Fig. 3:**
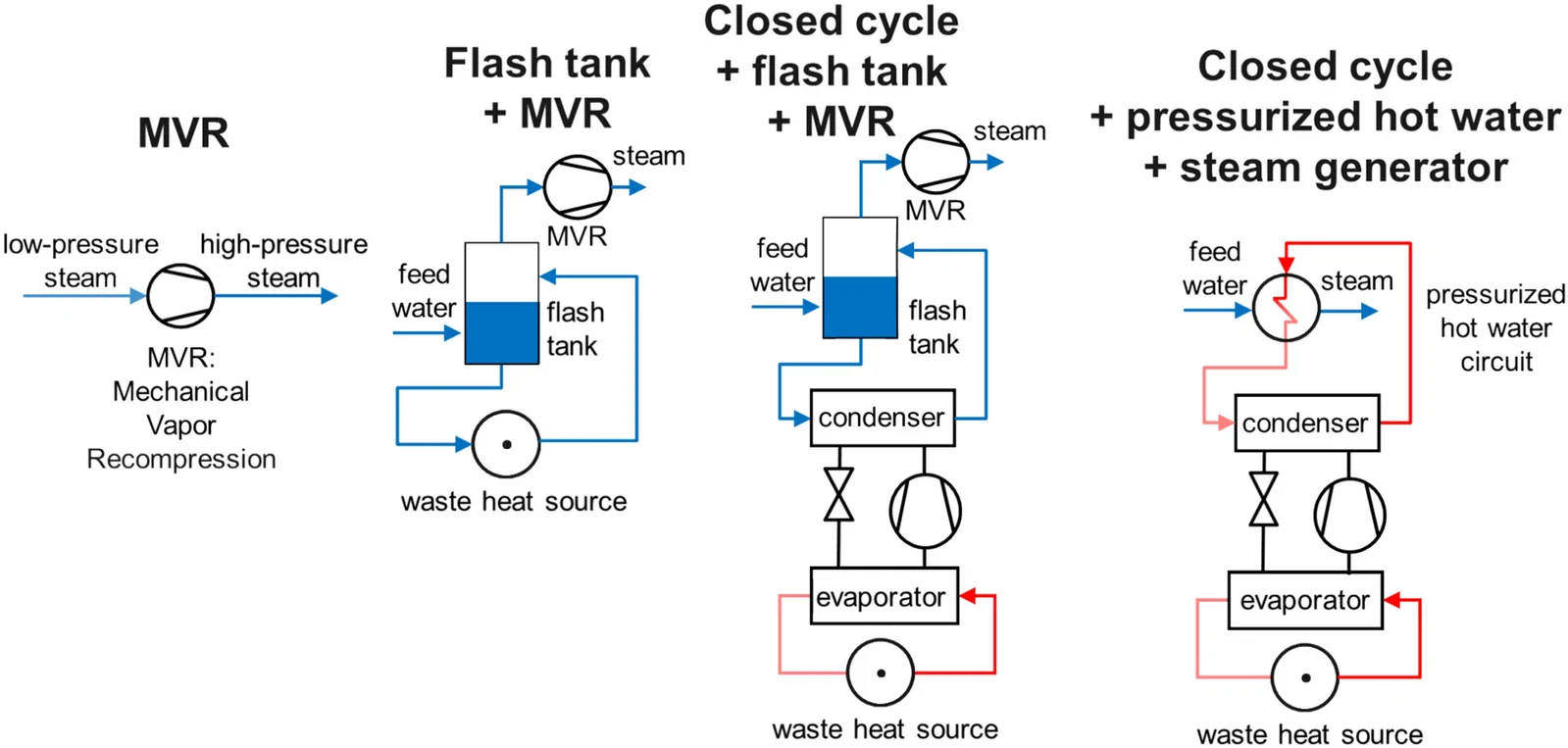
Heat pump concepts to generate steam (Arpagaus 2025a, 2025b)

For steam generation, mechanical vapor recompression (MVR) or a combination of high-temperature heat pumps (HTHPs), flash tanks/evaporators, and steam compressors is commonly used. As shown in Fig. 3, there are four main heat pump concepts for steam production (Arpagaus 2025a, 2025b):

- Mechanical vapor recompression (MVR) (steam compressors)
- MVR (steam compressors) combined with a flash tank
- Closed-cycle vapor compression heat pump with flash tank and MVR (steam compressors)
- Closed-cycle vapor compression heat pump with a direct heat exchanger for steam generation

### Refrigerants

**Fig. 4:**
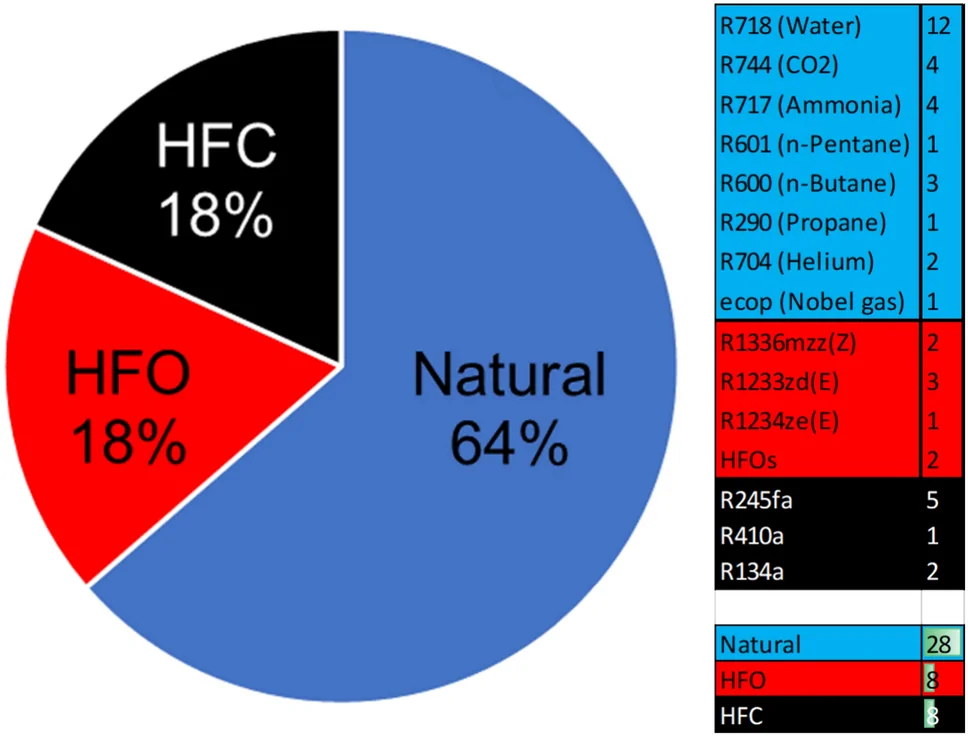
Distribution of refrigerants used in industrial HTHPs showing predominance of natural refrigerants (Arpagaus 2025a)

Figure 4 illustrates the distribution of refrigerants used within the HTHP market overview (2024) presented in Fig. 1 (Arpagaus 2025a). Natural refrigerants, including ammonia, CO_2_, hydrocarbons (such as propane, butane, and pentane), helium, and water, account for 64% of the HTHP products. Synthetic hydrofluoroolefins (HFOs) and hydrofluorocarbons (HFCs) each account for 18% of the market share.

Refrigerant selection increasingly focuses on both environmental performance and regulatory compliance. Natural refrigerants are favored for their low GWP and minimal environmental persistence. HFOs, while offering reduced GWP, face growing regulatory uncertainty due to their potential classification as PFAS (per- and polyfluoroalkyl) generating substances.

In the EU and other regions, regulatory bodies are reviewing whether HFOs should be restricted or banned, given their fluorinated structure and environmental persistence. This could significantly limit or phase out the use of HFO in future industrial heating and cooling applications. Consequently, industries such as the pharmaceutical sector often require natural refrigerants for new heat pump and chiller systems. Thus, manufacturers are proactively developing hydrocarbon-based or other natural refrigerant solutions to anticipate evolving chemical and PFAS regulations.

## Techno-economic considerations

**Fig. 5:**
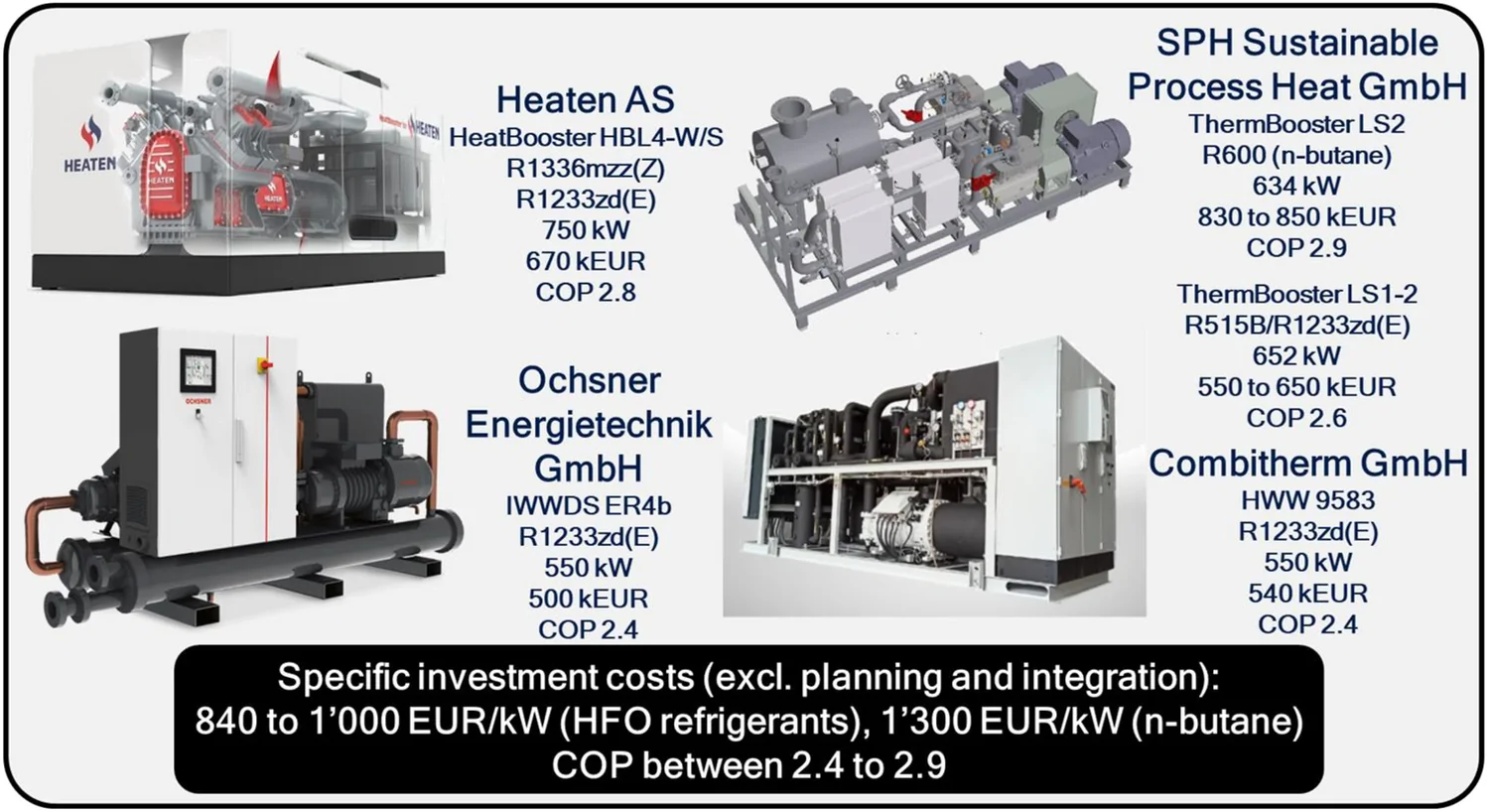
Specific investment costs (excluding planning and integration) of some steam-generating heat pumps (Arpagaus et al. 2024)

Capital cost is a major factor in evaluating the feasibility of steam-generating heat pumps. Figure 5 illustrates the specific investment costs (excluding planning and integration) for selected steam-generating heat pumps (Arpagaus et al. 2024). The specific investment costs range from EUR 840–EUR 1300 per kW of heating capacity, depending on the size and refrigerant choice (e.g., HFO or butane). Heat pump equipment accounts for approximately 30–50% of total project costs, depending on the integration complexity. Integration approaches range from boiler replacement at the supply level to waste-heat recovery at the process level.

The COP ranges from 2.4 to 2.9 for low-pressure steam applications, with a temperature lift of approximately 70 K. With a COP of 3, HTHPs offer a favorable life-cycle cost balance, as they consume only one-third of the electricity required by electric boilers, thereby reducing electrical grid capacity requirements. However, the ratio between electricity and gas prices is crucial. In markets with low gas prices and high electricity prices, COPs above 4 are required to achieve both economic and environmental benefits.

## Demonstration projects of industrial HTHPs in Europe

Several European projects are successfully demonstrating advanced heat-upgrade technologies for industrial process heat. Among the most prominent are the SPIRIT and Push2Heat projects, both funded under the Horizon Europe framework (HORIZON-CL5-2021-D4-01-04) (Push2Heat 2022; SPIRIT 2022). These projects aim to demonstrate the reliability, scalability, and decarbonization potential of different heat-upgrade technologies with a supply temperature range of 90–160 °C across sectors such as food, paper, and sugar manufacturing.

**Fig. 6:**
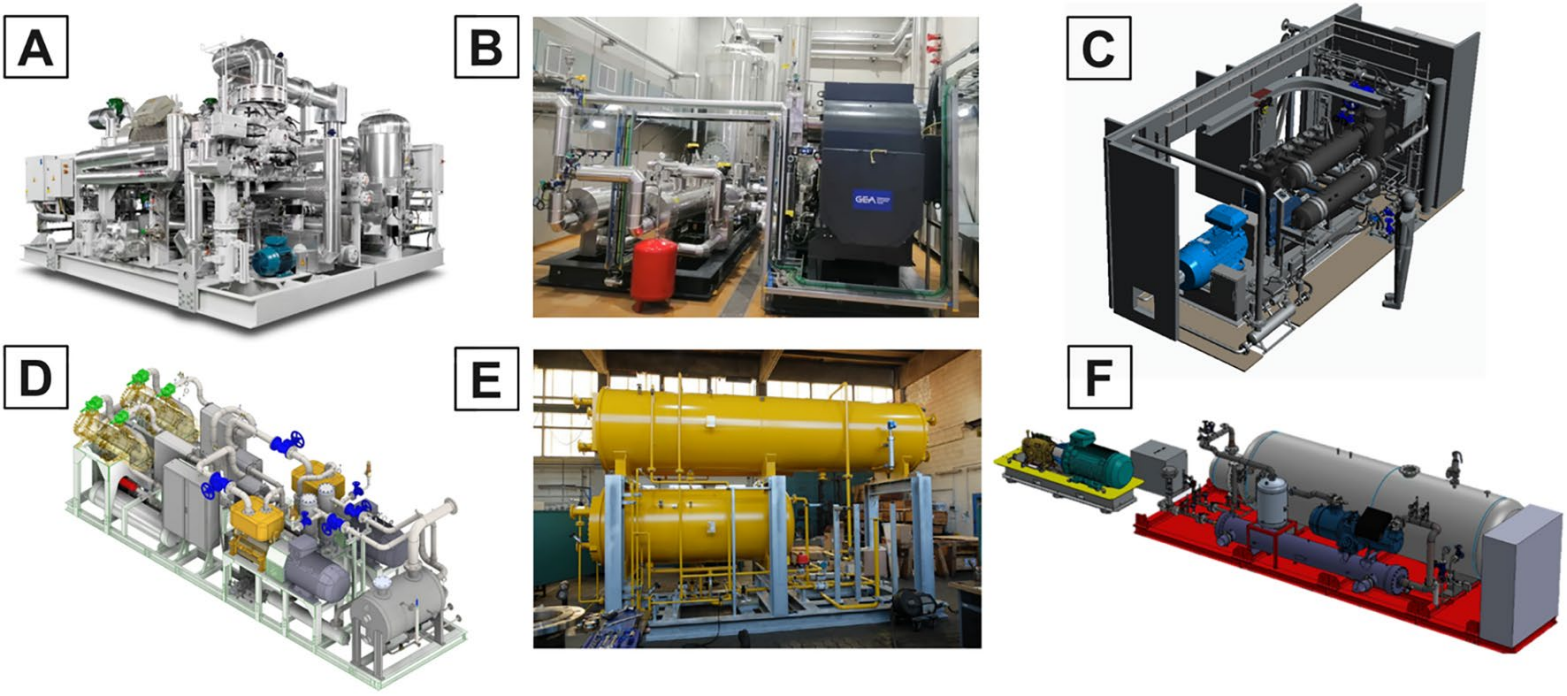
Demonstration projects: **A** Prototype of a pentane HTHP by Mayekawa for steam production, **B** GEA pentane HTHP installed at the Tiense Suiker sugar factory, **C** Four-cylinder piston steam compressor by Spilling in a container setup, **D** SPH Thermbooster at Felix Schoeller, **E** Absorption heat transformer by BS Nova at Cartiere di Guarcino, **F** HTHP with screw compressor by Cannon Bono at Cartiere di Guarcino (Data sources Arpagaus (2025a, 2025b, 2025c))

Figure 6 illustrates the developed heat-upgrade technologies. Table 1 provides an overview of the demonstration sites with key technical data, organized by application, heat-upgrade technology, technology provider, refrigerant, heat sink, heat source, and heating capacity.

**Table 1:** Overview of key data from demonstration projects on heat-upgrade technologies within the EU-funded SPIRIT and Push2Heat projects

Demo site (Country)	Stella polaris (Norway)	Tiense suiker (Belgium)	Smurfit kappa (Czech Republic)	Felix schoeller (Germany)	Cartiere di Guarcino (Italy)	Cartiere di Guarcino (Italy)
EU-project	SPIRIT demo 1	SPIRIT demo 2	SPIRIT demo 3	Push2Heat demo 1	Push2Heat demo 2	Push2Heat demo 3
Application	Prawn processing	Sugar production	Pulp and paper, paper drying	Paper drying process	Paper industry	Paper industry
Heat-upgrade technology	HTHP with screw compressors (2-stage design)	HTHP with screw compressor	MVR (4-cylinder steam compressor)	HTHP cascade with 2 screw and 2 piston compressors	Absorption heat transformer (thermally driven)	HTHP with screw compressor+MVR
Technology provider	Mayekawa	GEA	Spilling	SPH	BS Nova	Cannon Bono
refrigerants	Ammonia/Pentane (R717/R601)	Pentane (R601)	Steam (R718)	R515B (1^st^ stage), R1233zd(E) (2^nd^ stage)	H_2_O/LiBr, R718 (steam)	R1233zd(E) (HTHP), R718 (steam) (MVR)
Heat sink	~ 143 °C and 4.2 bar(a) steam	~ 139 °C and 3.8 bar(a) steam (or 114 °C depending on campaign)	~ 158 °C and 6 bar(a) steam	~ 124 °C and 2.85 bar(a) steam	133 °C and 3.3 bar(a) steam (~ 340 kW), 158 °C and 6.5 bar(a) steam recompressed by TVR using 14.5 bar steam	120 °C and 1.7 bar(a) steam (~ 740 kW), 158 °C and 6.5 bar(a) steam recompressed by MVR (~ 855 kW)
Heat source	20–80 °C (waste heat from cooling system, ~ 600 kW)	75–92 °C (vacuum steam from the evaporation plant)	~ 2 bar(a) (flashed condensate after the drying process)	~ 45 °C (exhaust air/water heat recovery, ~ 680 kW)	90 °C (cooling water of CHP plant, ~ 715 kW)	90 °C (~ 600 kW)
Heating capacity	~ 1.2 MW	~ 4 MW	~ 0.7 MW	~ 1.18 MW	~ 0.34 MW	~ 0.855 MW

At Stella Polaris in Norway, a Mayekawa MYCOM screw-compressor-based HTHP using ammonia and pentane in a two-stage design is integrated into a shrimp processing facility. The system delivers 1.2 MW of steam at 143 °C, replacing steam previously generated from fossil fuels. It recovers approximately 600 kW of waste heat from the existing cooling system, with dedicated safety measures in place to manage the use of the flammable refrigerant. If successful, the demonstration will reduce primary energy use by about 1000 MWh and CO_2_ emissions by roughly 600 tons per year.

At the Tiense Suiker sugar factory in Belgium, a GEA HTHP with a screw compressor system was installed to produce 4 MW of steam at 139 °C for sugar extraction, evaporation, and crystallization. The HTHP uses vacuum steam from the evaporation plant, ranging from 75 to 92 °C, as a heat source to generate process steam at approximately 139 °C. By replacing part of the steam previously produced by natural gas boilers, the system contributes to the partial electrification of the production process and reduces CO_2_ emissions by 3000–3500 tons annually.

At Smurfit Kappa in the Czech Republic, a 4-cylinder piston steam compressor from Spilling Technologies was integrated into a corrugated cardboard packaging plant to recover and compress excess low-pressure flash steam from condensate expansion. The system handles up to 1.0 t/h of steam (~ 700 kW), compressing it from around 2–6 bar(a) and 180 °C. This MVR unit is designed as a standardized, containerized system and serves as a prototype for future serial production. The demonstration aims to enable modular standardization of compressor units, reducing costs and improving process efficiency.

At the Felix Schoeller site for specialty paper production in Germany, a two-stage cascade HTHP system from SPH (consisting of two screw and two piston compressors in parallel) was integrated, using the synthetic refrigerants R515B and R1233zd(E) as working media. Due to safety considerations regarding the electrical transformer systems in the immediate vicinity of the installation site, the use of non-flammable refrigerants was required. The system delivers 1.18 MW of saturated steam at 123 °C and achieves a COP of 2.3–2.5. The system reduces natural gas use, saving approximately 1200 tons of CO_2_ annually.

At Cartiere di Guarcino (Italy), BS Nova implemented a thermally driven absorption heat transformer (AHT) using H_2_O/LiBr working fluid. The system utilizes 90 °C waste heat from the CHP plant’s cooling water (~ 715 kW) as the driving source to produce approximately 0.34 MW of steam at 3.3 bar(a). This steam is subsequently recompressed to 6.5 bar(a) using 14.5 bar(a) motive steam in a thermal vapor compressor (TVC) unit.

A second demonstrator at Cartiere di Guarcino (Italy) is an HTHP by Cannon Bono, using refrigerant R1233zd(E) and a screw compressor. The system recovers 600 kW of waste heat from the cogeneration plant’s cooling water (90–80 °C) to produce 1.7 bar(a) steam (115 °C, ~ 740 kW). This steam is then recompressed via MVR to 6.5 bar(a) (162 °C, ~ 855 kW) and injected into the medium-pressure steam distribution system.

## Implications, challenges, and opportunities for advancing HTHP adoption

HTHPs represent a critical pathway for the decarbonization of industrial process heat, addressing the Special Issue’s focus on scalable, low-carbon solutions for energy-intensive industries. The review highlights that, while HTHPs are technically capable of supplying heat up to ~ 280 °C.

The economic feasibility of HTHPs depends mainly on electricity tariffs, capital costs, grid capacity, and operational models. With typical COPs of 3–4, HTHPs reduce electricity demand by roughly a factor of three compared to direct electric heating. Hybrid systems that incorporate thermal storage and flexible operation during periods of low electricity costs further optimize economics.

However, several key challenges remain for widespread adoption, such as:

- high upfront investment costs
- limited stakeholder knowledge
- volatility in electricity prices and carbon intensity
- integration complexity into existing industrial processes
- constraints on refrigerant selection, and
- operational reliability under continuous industrial loads.

These factors can limit the pace of HTHP deployment despite its demonstrated efficiency and CO_2_-reduction potential.

Enablers include supportive policies, demonstration projects (e.g., SPIRIT and Push2Heat), and the integration of onsite renewables with thermal storage. Together, these factors can accelerate HTHP adoption across industrial sectors, supporting decarbonization and sustainability targets.

At the same time, the review identifies substantial opportunities. European demonstration projects and case studies indicate that HTHPs can deliver substantial reductions in process heat-related emissions, while market evolution data show an increasing number of suppliers and system innovations, signaling growing technological maturity.

Key recommendations for advancing the adoption of HTHPs in industrial applications include:

1. *Enhance System Integration:* HTHPs should be closely integrated with existing industrial processes to optimize energy recovery and minimize operational costs. This includes aligning HTHP operations with process heat demands and effectively leveraging waste-heat sources.
2. *Standardize Components:* There is a need for standardized, modular components to reduce costs and facilitate scalability. This approach can also simplify maintenance and improve system reliability across various industrial sectors.
3. *Optimize Refrigerant Selection:* Selecting appropriate low-GWP refrigerants is crucial for enhancing system efficiency and reducing environmental impact. The study emphasizes the importance of considering both thermodynamic performance and safety aspects when choosing refrigerants.
4. *Implement Hybrid Systems:* Combining HTHPs with thermal energy storage and renewable energy sources can enhance system flexibility and economic viability. Hybrid systems enable operation during off-peak electricity hours, facilitating better integration with renewable energy sources and resulting in cost savings and reduced carbon emissions.
5. *Support Policy, Knowledge and Technology Transfer, and Dissemination:* To accelerate adoption, supportive policies, financial incentives, and knowledge-sharing platforms are essential. Demonstration projects and case studies can showcase the benefits and feasibility of HTHPs, encouraging wider implementation.

These recommendations aim to address the economic and technical barriers to HTHP adoption and to facilitate their role in industrial decarbonization strategies.

## Excursus: advancing HTHP adoption in New Zealand’s food sector

**Fig. 7:**
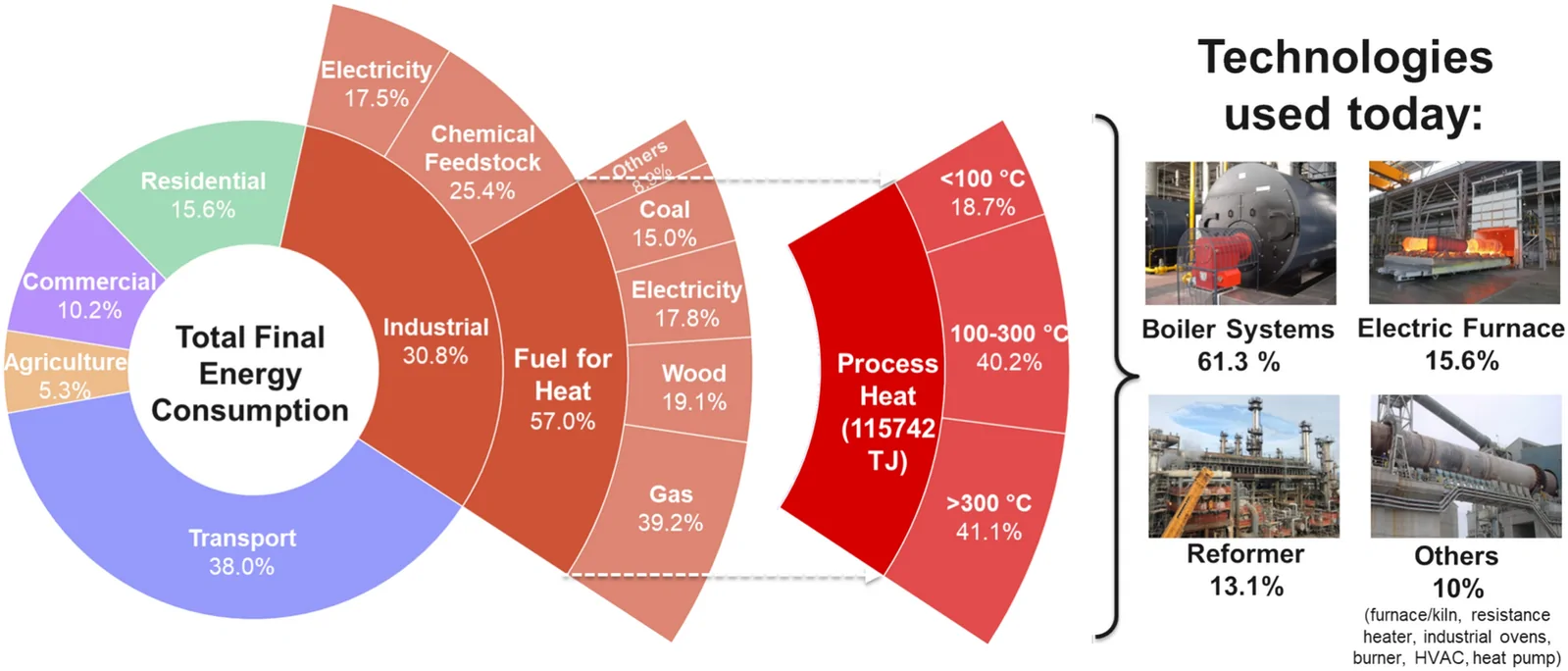
Industrial process heat demand in New Zealand by sector, energy carrier, temperature range, and technology used today (Arpagaus (2025a), data based on EECA (2024))

**Fig. 8:**
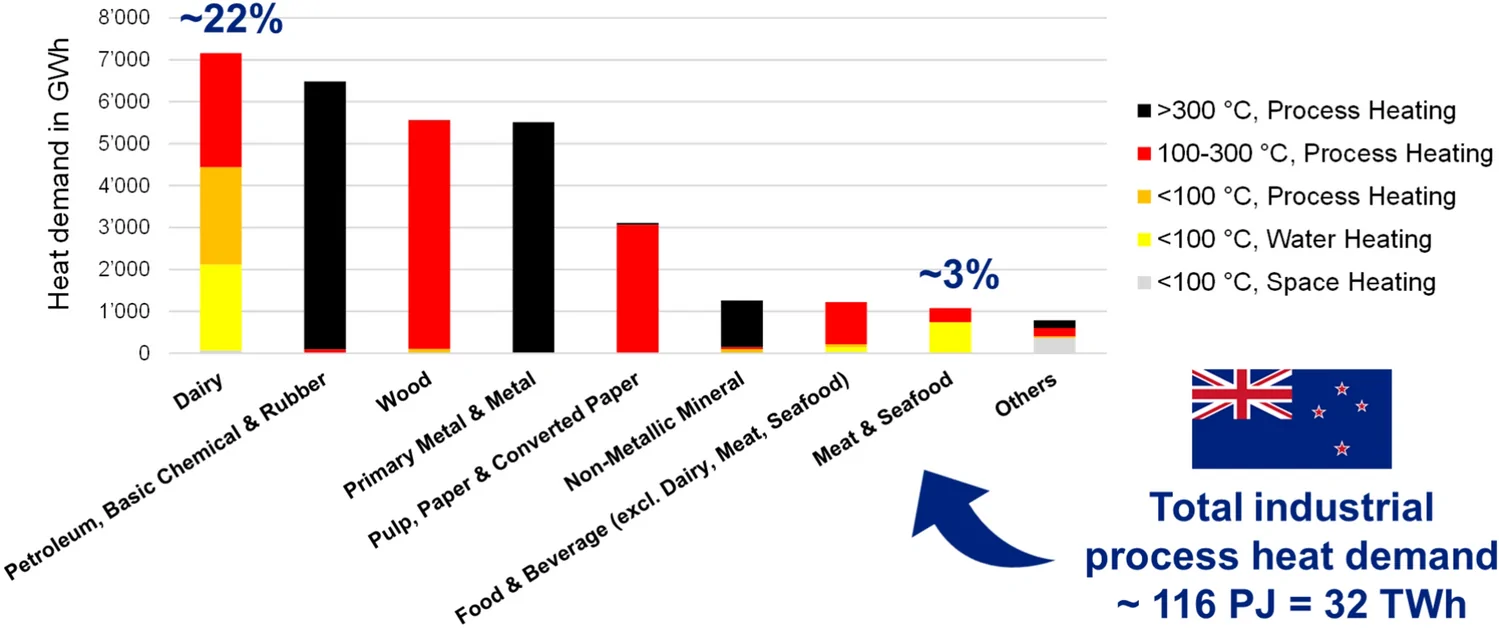
Industrial process heat demand in New Zealand (Arpagaus (2025a), data based on EECA (2024))

In New Zealand, the industrial process heat demand accounts for around 116 PJ (32 TWh) and is concentrated in the dairy, meat, petroleum, wood, and metals sectors, with a significant share between 100 and 300 °C (EECA 2024) (Figs. 7, 8). Currently, over 60% of this heat is supplied by fossil-fuel boilers, while heat pumps account for less than 1% of industrial heat use.

**Fig. 9:**
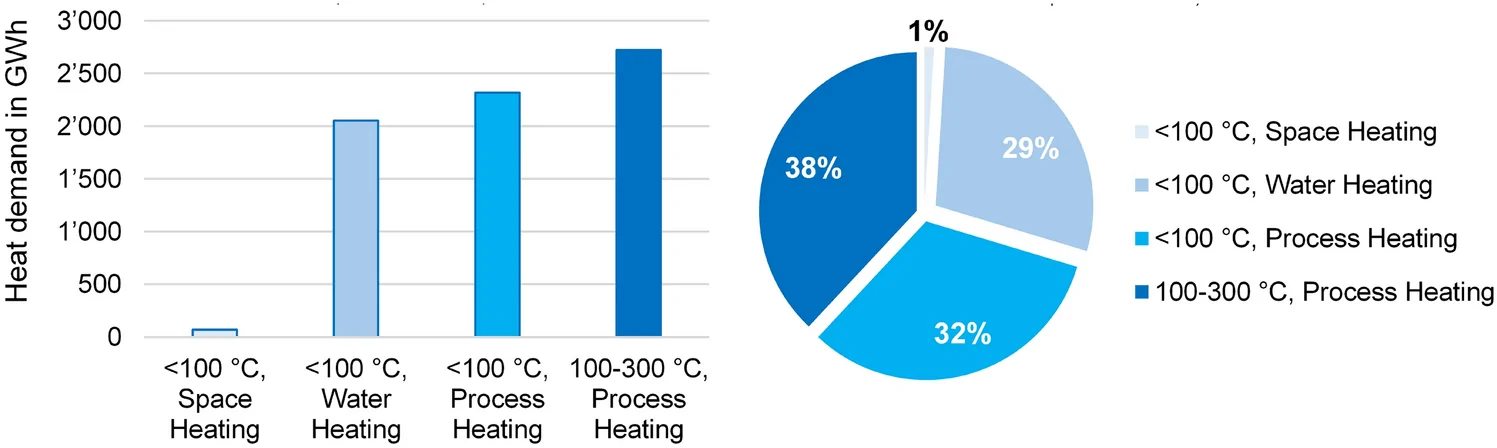
Industrial process heat demand in New Zealand’s dairy sector (dairy cattle farming and dairy process manufacturing) (Arpagaus (2025a), data based on EECA (2024))

The dairy industry (Fig. 9), which relies on large milk powder spray dryers requiring steam at 120–200 °C, presents significant potential for HTHPs (around 22% of New Zealand’s total process heat demand), particularly for preheating drying air, milk concentration, and integration with Mechanical Vapor Recompression (MVR).

**Fig. 10:**
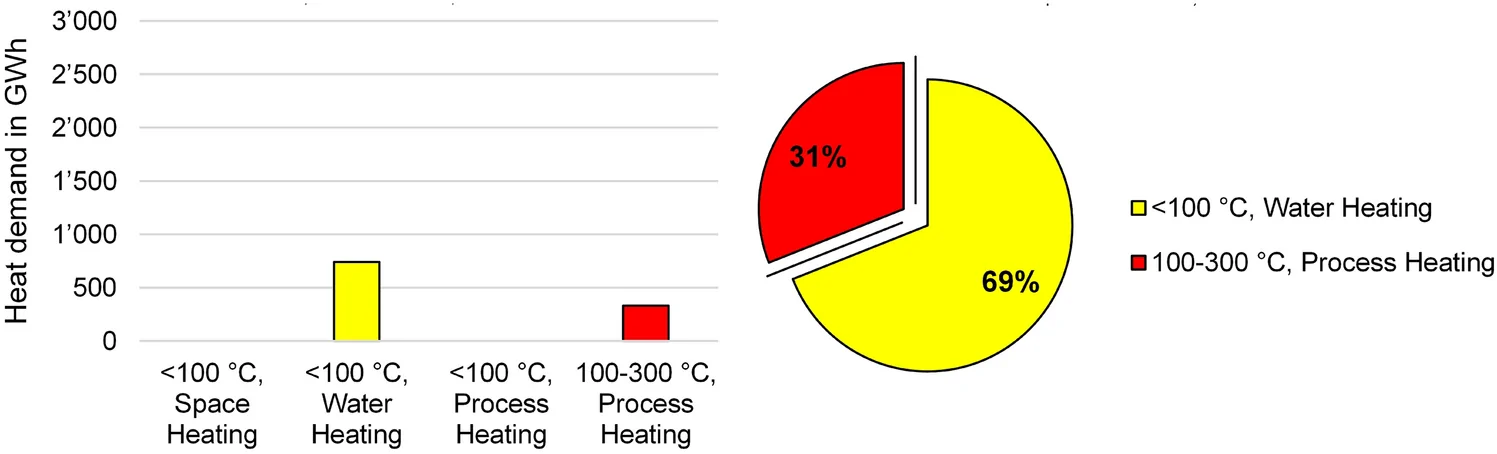
Industrial process heat demand in New Zealand’s meat sector (meat and meat product manufacturing and seafood) (Arpagaus (2025a), data based on EECA (2024))

Meat processing (Fig. 10), which demands large amounts of hot water (60–90 °C) for sterilization and cleaning, is well-suited for water-to-water ammonia heat pumps. Other sectors, such as petroleum refining and wood processing, also offer opportunities for integrating hybrid HTHP and MVR, particularly where abundant waste heat is available.

Waste heat from cooling-water systems can be efficiently recovered, and HTHPs can preheat amine regenerators, reducing steam demand. MVR is well-suited for evaporation, distillation, and vapor recompression processes, especially when handling large vapor volumes. Kraft pulp mills are highly steam-intensive and generate significant waste heat. MVR is already established in black-liquor evaporation, while HTHPs can recover waste heat from evaporators and effluent streams to provide heat for bleaching and pulp washing. Paper machines have large, continuous thermal loads; HTHPs can upgrade dryer-exhaust heat to steam.

The analysis highlights that the food sectors share similarities with Swiss industrial heat profiles, making HTHP solutions highly transferable.

**Fig. 11:**
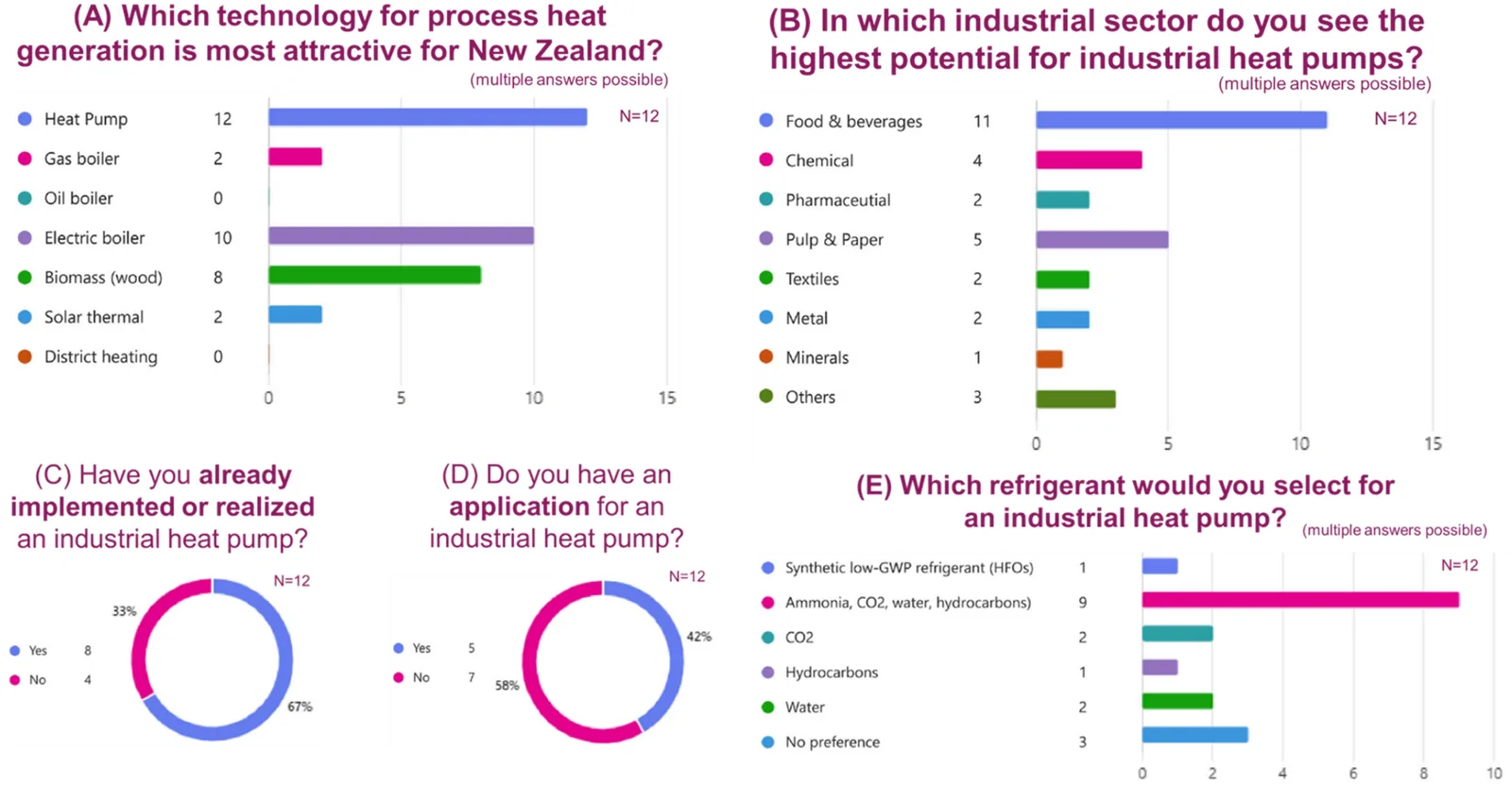
Survey results presented at the EECA workshop (energy efficiency & conservation authority) in Auckland, New Zealand, 24 February 2025 (Arpagaus, 2025c)

In addition, Fig. 11 shows the results of a survey conducted with 12 participants at the EECA Workshop (Energy Efficiency & Conservation Authority) in Auckland on 24 February 2025 (Arpagaus, 2025c). The food and beverages sector was identified as having the highest potential for industrial heat pump applications. Alternative heating technologies included electric boilers and biomass (wood). For refrigerant selection, ammonia and other natural refrigerants were preferred for industrial heat pump applications.

Advancing HTHP adoption in New Zealand requires consideration of several system-level and market factors (Atkins et al. 2025). Future industrial utility systems are expected to increasingly rely on electricity, biomass, or hybrid boiler configurations. Analysis of industrial process heat flows highlights potential low-temperature heat demand below 100 °C, offering promising integration opportunities for HTHPs.

Levelized cost of energy (LCOE) assessments for HTHP cases ranging from 50 °C heat source upgraded to 120 °C (2 bar steam, COP of 2.8) and from 40 to 144 °C (4 bar steam, COP of 2.0) indicate that fuel price, particularly the cost of electricity, is the dominant driver of LCOE. Under current price conditions, HTHP-generated heat can be cost-competitive with biomass and coal (Atkins et al. 2025). LCOE of HTHPs is significantly lower than that of electrode boilers. Coal-fired systems are expected to be banned by 2037, and major gas supply constraints are anticipated, further strengthening the strategic value of electrified heating technologies.

Demonstration plants in New Zealand would accelerate market confidence and build local capability. Major HTHP suppliers, including GEA, Everllence, Mayekawa, Mitsubishi Heavy Industries, Johnson Controls, and others, could play a critical role in scaling deployment and providing local support. EECA has also published best-practice guidelines for HTHPs to support installers, engineers, and facility managers in selecting and integrating HTHPs across residential, commercial, and industrial applications (EECA 2025).

Key challenges for HTHP technology include waste-heat and heat-source availability, temperature requirements, and scaling HTHP to the MW range.

Key technical challenges include the availability of waste heat and heat sources at the right temperature, meeting required temperature lifts, scaling HTHP solutions to the MW-levels, and proper integration into industrial processes (Atkins et al. 2025).

## Conclusions and outlook

Overall, the findings emphasize that high-temperature heat pumps (HTHPs) are not just a niche technology but a scalable lever for industrial decarbonization, offering concrete pathways to meet net-zero targets while supporting the broader transition to electrified, low-carbon industrial energy systems. This is precisely the strategic focus of the Special Issue.

HTHPs are a technologically mature and commercially viable solution for industrial decarbonization, capable of providing heat up to around 280 °C using steam compressors. Demonstration projects such as SPIRIT and Push2Heat in Europe confirm their technical feasibility and operational efficiency, with immediate application potential in the food & beverage, chemical, and pulp & paper industries.

Steam-generating heat pumps (SGHPs) show strong potential for low-pressure steam applications, leveraging established large-scale compressor technologies and ongoing innovations in small- to medium-scale systems (e.g., oil-free, high-speed turbo, and multi-stage compression).

Operational demonstrations indicate significant energy savings and average COPs of around 3.2 at a 62 K temperature lift, underscoring HTHPs as a key technology for sustainable industrial energy strategies. However, market adoption remains constrained by the electricity-to-gas price ratio, high investment costs, and a lack of industrial-scale demonstration projects, particularly in regions such as New Zealand and others dominated by fossil-based process heat. The New Zealand food sector excursus illustrates how regional adaptation and sector-specific pathways can accelerate deployment, highlighting transferable strategies for other regions and industries.

A growing number of industrial HTHP products are now available, employing concepts such as direct evaporation, flash evaporation, steam compressors, and combined cycles. The most common business model remains direct-to-customer ownership, encompassing sales, installation, commissioning, maintenance, and service contracts.

Looking forward, the widespread adoption of HTHPs will depend on advancements in compressor technology, the use of natural and low-GWP refrigerants (e.g., H_2_O, CO_2_, NH_3_, hydrocarbons, helium, HFO/HCFOs), cost reductions, and supportive policy frameworks.

Early demonstration projects in fossil-fuel-intensive sectors, particularly those in dairy and meat processing, could catalyze broader market adoption. In the long term, progress in multi-stage, oil-free, high-speed compression, optimized refrigerant blends, and scalable service-oriented business models will further enhance system efficiency, reliability, and sustainability, positioning HTHPs as a central pillar of global industrial decarbonization.

## Data Availability

No datasets were generated or analyzed during the current study.
